# Fast and Simple Molecular Test for Sex Determination of the Monomorphic *Eudromia elegans* Individuals

**DOI:** 10.3390/ani14121719

**Published:** 2024-06-07

**Authors:** Zuzana Majchrakova, Marcela Bielikova, Evelina Hrckova Turnova, Petra Gasparkova, Jan Turna, Andrej Dudas

**Affiliations:** 1 Slovgen Ltd., Ilkovicova 8, 841 04 Bratislava, Slovakia; majchrakova@slovgen.sk (Z.M.); gasparkova@slovgen.sk (P.G.); 2 Department of Molecular Biology, Faculty of Natural Sciences, Comenius University, Ilkovicova 6, 842 15 Bratislava, Slovakia; marcela.bielikova@uniba.sk (M.B.); jan.turna@uniba.sk (J.T.); 3 Comenius University Science Park, Ilkovicova 8, 841 04 Bratislava, Slovakia; evelina.hrckova.turnova@uniba.sk

**Keywords:** *Eudromia elegans*, sex determination, PCR, primers

## Abstract

**Simple Summary:**

A lot of avian species are morphologically monomorphic, and thus, sex determination is difficult if it is based only on their external examination. Molecular sex identification has become a crucial tool in various fields of biology, including ornithology. This non-invasive method used to determine the genetic sex of birds of any age, in our case, *Eudromia elegans* (Paleognathae, Tinamiformes), can proceed with regard to the differences between avian male (ZZ) and female (ZW) chromosomes. According to our experience, the other molecular methods failed in the sex determination of this exact species. The new test was designed to be reliable, fast, and simple to determine the sex of the individuals of *E. elegans*. Molecular sexing has significant implications for understanding various aspects of its biology, including mating systems, population genetics, and conservation strategies. By accurately determining the sex of individuals within a population, researchers can investigate sex ratio patterns, reproductive success, and gene flow.

**Abstract:**

Sex determination based just on morphological traits such as plumage dichromatism, sexual size dimorphism, behavior, or vocalizations is really challenging because of the sexual monomorphism present in more than half of avian species. Currently, a lot of them can be tested through DNA-based procedures, but they do not fit all the avian species, such as *Eudromia elegans*. The aim of this study was to design a new molecular method suitable for routine sex determination for that species that is fast, simple, and cost- and time- effective. DNA was isolated from dry blood stain and/or chest feather samples of *E. elegans* species. We used two sets of sex-specific primers (ZF/ZR and WF/WR) to amplify the expected fragments localized on the highly conserved *CHD1* gene to distinguish between sexes due to the W-specific DNA sequence present only in females. We confirmed the accuracy and consistency of the PCR-based method based on length differences to distinguish between the sexes of *E. elegans*, which amplified two fragments in females and one fragment in males.

## 1. Introduction

All modern birds, with their species richness and global distribution, from the smallest bee hummingbird to the largest ostrich, can be divided into two large groups based on their bony palatal structure: the Palaeognathae (flightless ratites and weak-flying tinamous) and the Neognathae (all other carinates). The Paleognathaes form a group of less than 1% of all the extant avian species. The mostly terrestrial pheasant-like Tinamiformes species (Palaeognathae), including *Eudromia elegans*, inhabit the tropical New World biogeographic region that extends south, east, and west from the central plateau of Mexico [1,2,3,4].

The majority of the avian species have 37 to 43 pairs of chromosomes (range for all birds: 20 to 63 pairs), including one pair of Z and W gonosomes. In contrast to mammals, where females are the homogametic sex (XX), and males are the heterogametic sex (XY), avian male individuals carry the homogametic ZZ chromosomes and female individuals the heterogametic ZW chromosomes [5,6].

Regardless of the ancient origin, the dynamics of genome changes and chromosome rearrangements in the Palaeognathae are still very low in comparison with other birds, which can explain the most primitive forms of avian sex chromosomes. They are broadly homomorphic, as cytogenetic studies on mitotic chromosomes have acknowledged [1,5,7,8].

Sex determination based solely on external morphology is a challenging task. This difficulty is not just an inconvenience for researchers; it has significant implications for various aspects of avian biology, including understanding mating systems, population genetics, and conservation strategies. Accurate sex determination within a population allows researchers to investigate patterns of sex ratio, reproductive success, and gene flow, which are crucial for an effective understanding of evolutionary dynamics, conservation management, and added advantages in forensic cases [9,10,11,12,13,14].

The non-invasive molecular procedures, especially the PCR method or Southern blotting, for avian sex determination in monomorphic species present very valuable, quick, accurate, and relatively simple tools. Over 50% of volant birds are monomorphic, showing no sexual dimorphic traits. In nestlings, the percentage is even higher, making molecular sexing methods highly necessary, especially for captive breeding management, commercial purposes, and conservation programs. Accurate sex determination is essential for understanding avian biology, including mating systems, population genetics, and conservation strategies. Molecular sexing methods, such as PCR, have become indispensable tools for such purposes, especially in monomorphic species where traditional methods are not reliable [15,16,17].

Here, we report a simple and improved DNA-based test for sex determination in *E. elegans*. To overcome the limitations of the other commonly used primers, we designed the two sets of primers (ZF/ZR and WF/WR) specific for the *CHD1* gene for the mentioned species, in which well-known primers [15,18,19] did not work in our experience.

## 2. Materials and Methods

Blood and feather samples were collected from the species *E. elegans*, also known as the elegant crested tinamou. Ten samples from a breeder (4 males, 4 females, 2 unknown sex) were used to optimize and implement the method. Biological material for DNA isolation was obtained from a bird’s foot (dry blood stain) and/or from the chest feathers (amount of 6–8 pcs) provided by owners. DNA isolation has proceeded via the manufacturer’s instructions. We used Chelex for DNA isolation from dry blood stains and Phire Animal tissue direct PCR kit (Thermo Fisher Scientific™, Waltham, MA, USA) for DNA isolation from the chest feathers. The primers used in this study were designed in the *CHD1* gene in the exonic region 6 for both forward primers and in the intron 6 for both reverse primers. The sequence of primers for two separate amplicons were: forward primers: WF—5′-TCTTGAAAGAGCCAAGAAGA-3′ and ZF—5′-TCTTGAAAGAGCCAAGAAGA-3′, reverse primers: WR—5′-CTAAAATATAGGAATTTCAGAATAAATC-3′ and ZR—5′-CTGAAACACAGGAATTTCAGA-3′. We used the sequence of *CHD1Z* and *CHD1W Eudromia elegans* from GenBank (AB255123 and AB255124) to design the primers. Two PCR amplifications were performed in separate 20 µL reaction mixtures, each containing approximately 50 ng of the DNA template, 2 µL 10× buffer (with the blue and yellow dye), 1.2 µL 25 mM MgCl_2_, 0.4 µL 12.5 mM dNTPs, and 0.4 µL of each primer. PCR reaction conditions were as follows: after 4 min of denaturation at 94 °C, 38 cycles were used with the following steps: 30 s at 94 °C, 45 s at 56 °C, 45 s at 72 °C, followed by the final stage of extension 8 min at 72 °C. The PCR products were separated on a 1.5% agarose gel in 0.5 × TBE at 120 V for 45–60 min. The gels were stained with ethidium bromide and visualized under ultraviolet light to confirm the presence of the expected amplicons.

## 3. Results

**Figure 1:**
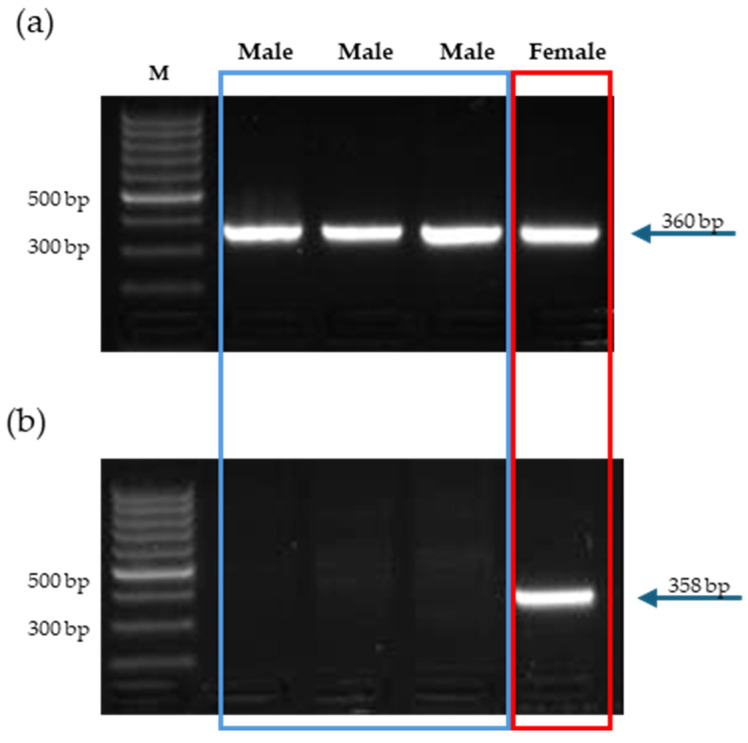
Electrophoretic gel separation showing results of the PCR reactions of sex determination of the *Eudromia elegans* individuals obtained with two primer sets: (**a**) PCR reaction using primers ZF/ZR, length of PCR product—360 bp; (**b**) PCR reaction using primers WF/WR, length of PCR product—358 bp. M = 100 bp DNA molecular size marker, blue rectangle—Male, fragment length 360 bp, red rectangle—Female, two fragments observed, length 360 bp and 358 bp.

In this study, the two pairs of primers (ZF/ZR and WF/WR) were used to successfully amplify the two fragments of the section of the *CHD1* gene, which is necessary to determine the gender of *E. elegans* individuals. The designed primers are not based on different sizes of introns as commonly used, e.g., P2/P8 or 2550/2718 [18,19], but on a different sequence of the mentioned section of the *CHDZ* and *CHDW* gene, thus two PCR reactions are needed, in contrast to one PCR reaction, which is sufficient in the other mentioned methods. The obtained size of the amplicons was WF/WR—358 bp and ZF/ZR—360 bp. PCR reactions were performed separately but under the same conditions. Figure 1 shows their analysis on a 1.5% agarose gel. The male characteristic DNA banding pattern indicated the presence of just one band in the PCR reaction, where the primers ZF/ZW were used, and the complete absence of the band in the other reaction (WF/WR), contrary to a female pattern where the presence of both fragments is visible to distinguish between the two sexes properly.

DNA sequencing was performed on the amplified products to confirm the specificity of our PCR assay. The obtained sequences were aligned with the *CHD1* gene sequences from *E. elegans* available in the GenBank database, confirming the accurate amplification of the target regions and validating the specificity of our designed primers for sex determination in *E. elegans*.

## 4. Discussion

Molecular techniques for sex determination have a lot of advantages, especially for monomorphic species such as *Eudromia elegans*, also known as the elegant crested tinamou. Traditionally, figuring out the bird’s sex relies on phenotypic characteristics in an adult stage of life (plumages, body size, radiographic morphometry, etc.) or fecal steroid analysis [17,20], which is not always reliable, or more invasive laparotomy laparoscopy, cloacal examination in species without apparent sexual dimorphism. The vent sexing method is usually in concordance with the results of the molecular technique’s determination, except in two birds. Therefore, there is a small possible space for errors in such a method [2,15].

*E. elegans* belongs to the neotropical family Tinamidae, the only living group of Palaeognathae able to fly, which is considered to be a sister group of the flightless ratites [1]. For this reason, we decided to focus on the primers w1/k7, which Huynen et al. described in 2002 as an efficient method to determine the sex in the avian ratite group [21], as well as the commonly used, independently developed primer sets 2550F/2718R and P2/P8 [18,19,22]. The analyses with the three known sets of primers (w1/k7, 2550F/2718R, P2/P8) showed limitations in accuracy and reliability. After the separation on the agarose gel electrophoresis, the results showed a lot of non-specific bands of various sizes, or the samples produced only male-specific fragments, even though we had expected that these results would not be probable.

At first, we tried to manage just one PCR reaction with all four primers (ZF, ZR, WF, and WR). The difference between two fragments occurred to be of such similar length, just 2 bp; hence, subsequent restriction digestion was needed using the *Dra*I restriction enzyme with a restriction site specific for the female-specific sequence of the *CHD1* gene. This was unnecessarily time and money consuming for such a routine analysis. With this in mind, we decided to perform separate PCR reactions to compensate for the length resemblance, with one set of primers in each. This polymerase chain reaction-based procedure successfully sexed all the individuals and has proven to be a reliable, simple, fast, solid, and cost-effective method for sex determination in *E. elegans*. The minor disadvantage of this protocol compared to the commonly used methods is that there is a need to perform two separate PCR reactions. Real-time PCR, which has been successfully used for sex determination [23,24,25], should eliminate the need for a second PCR reaction as long as it allows product quantification. On the other hand, it requires specific laboratory equipment.

The development and optimization of molecular techniques for sex determination are crucial for both conservation and breeding programs. The method we employed builds upon the work of previous studies, enhancing the accuracy and reliability of sex determination in *E. elegans* [2,15,18]. Furthermore, the simplicity and cost-effectiveness of our PCR-based procedure can be beneficial for studies involving other avian species, especially those with limited genomic resources or in field settings.

## 5. Conclusions

In conclusion, this study presents a new and improved DNA-based test for sex determination in the monomorphic elegant crested tinamou, *Eudromia elegans*. The described technique is based on using two sets of primers (ZF/ZR—360 bp and WF/WR—358 bp) under the same PCR conditions. Amplified fragments are separated by agarose gel electrophoresis. The method can be used to clearly distinguish between males and females, which allows fast, accurate, and cost-effective sexing of these birds. The use of two sets of primers specific to the *CHD1* gene allowed for accurate and reliable sex determination, overcoming the limitations of previously used primers. This PCR-based method offers a valuable tool for researchers working with monomorphic avian species. It has important implications for understanding various aspects of avian biology, including mating systems, population genetics, and conservation strategies. Future studies could explore the applicability of this method to other monomorphic avian species and investigate its potential for broader conservation and management applications.

The satisfactory use of the studied primers seems to be promising for the purposes of sex determination in all the individuals belonging to the order Tinamiformes.

## Data Availability

Data supporting this study are available from Z. Majchrakova upon reasonable request.
